# Gaps and Opportunities in HIV Service Delivery in High Volume HIV Care Centers in Liberia: Lessons From the Field

**DOI:** 10.5334/aogh.3246

**Published:** 2021-11-24

**Authors:** Mukhtar A. Adeiza, Ian Wachekwa, Cecilia Nuta, Sean Donato, Freda Koomson, Jane Whitney, Chelsea Plyler, Lila Kerr, Godsway Sackey, Elizabeth Dunbar, Kristina Talbert-Slagle, Robin Klar, Regan H. Marsh, Samretta Caldwell, Julia Toomey, Onyema Ogbuagu

**Affiliations:** 1 Yale School of Medicine, John F. Kennedy Medical Center, US; 2 John F. Kennedy Medical Center, LR; 3 UNICEF Supply Division, DK; 4 Yale School of Medicine, University of Liberia, Redemption Hospital, US; 5 Yale School of Medicine, US; 6 Brigham and Women’s Hospital, Partners In Health, US; 7 Brigham and Women’s Hospital, Partners In Health, LR; 8 Resilient and Responsive Health System, University of Washington, US; 9 New York University Rory Meyers College of Nursing, US; 10 Liberia National AIDS and STI Control Program, LR

## Abstract

**Background:**

Human Immunodeficiency Virus (HIV) infection continues to have a profound humanitarian and public health impact in western and central Africa, a region that risks being left behind in the global response to ending the AIDS epidemic. In Liberia, where the health system is being rebuilt following protracted civil wars and an Ebola virus disease outbreak, the Resilient and Responsive Health System (RRHS) is assisting with quality HIV services delivery through support from PEPFAR and HRSA but gaps remain across the cascade of care from diagnosis to viral load suppression.

**Objective:**

To highlight gaps in HIV service delivery in Liberia, identify opportunities and offer recommendations for improving the quality of service delivery.

**Methods:**

A narrative review of relevant literature was conducted following a search of all local and online databases known to the authors.

**Findings:**

Antiretroviral therapy (ART) has transformed the HIV response in Liberia by averting deaths, improving quality of life, and preventing new HIV infections but critical gaps remain. These include weak HIV prevention and testing strategies; suboptimal ART initiation and retention in care; low viral load testing volumes, commodity supply chain disruptions and a HIV workforce built on non-physician healthcare workers. In the context of the prevailing socioeconomic, heath system and programmatic challenges, these will impact achievement of the UNAIDS targets of 95-95-95 by 2030 and ending the epidemic.

**Conclusion:**

Combination prevention approaches are necessary to reach the most at risk populations, while a robust health workforce operating through facilities and communities will be needed to reach people with undiagnosed HIV earlier to provide efficient and effective services to ensure that people know their HIV status, receive and sustain ART to achieve viral suppression to maintain a long and healthy life within the framework of overall health system strengthening, achieving universal health coverage and the sustainable development goal.

## Background

The Joint United Nations Program on HIV/AIDS (UNAIDS) estimated that 38 million people worldwide were living with HIV at the end of 2019, with 25.7 million of those residing in sub-Saharan Africa [1]. While the number of new infections and AIDS-related deaths have decreased globally since 2013, West and Central Africa still accounted for 21% of new infections and 30% of global deaths (2017) [2]. The observed global decline in HIV cases was driven by disease control efforts in the general population although key and/or vulnerable populations such as adolescents, cisgender women, commercial sex workers (CSW), men who have sex with men (MSM) and prisoners are often neglected in HIV prevention efforts especially in resource-limited settings [1,2]. In Liberia, in 2017, it was estimated that 40 000 people were living with HIV, including around 3 000 children aged 0–14 years. Unfortunately, fewer than one in three reproductive age adults (15–49 years) who are living with HIV had access to medicines to treat their HIV infection both for their own benefit and to reduce disease transmission [3].

While there have been remarkable scientific and programmatic advances in HIV treatment and prevention, a cure remains elusive, and despite valiant efforts by public health authorities such as the World Health Organization, Global Fund for AIDS, Tuberculosis and Malaria, as well as the US President’s Emergency Plan for AIDS relief that have formed the backbone of policy, guidelines and resources for AIDS care in Africa, too many people with HIV or at risk for HIV still do not have access to prevention, care, and treatment. Beyond the health effects of HIV, the epidemic has wide ranging negative impact on households, communities, and economies of countries in the world that can least afford to deal with the public health threat [2]. Many of these countries hardest hit by HIV also have to deal with other infectious diseases, food insecurity, and recently, an exploding burden of non-communicable diseases (NCDs). Stigma and discrimination, together with other social inequities and exclusion, remain key barriers to HIV-related efforts. Furthermore, the recent and ongoing 2020 COVID-19 pandemic has seriously impacted HIV programs and could disrupt it even more in the months to come [4].

## HIV in Liberia- Stats and Figures

The HIV prevalence in Liberia ranges from 1.5% to 2.1% among people 15–49 years of age [4,5] with women having a higher prevalence of 2.4% compared to men among whom it is 1.9%, not uncommon in SSA [5]. Significant variations in HIV prevalence exist being higher in urban (2.6%) than in rural (0.8%) areas [5]. The South-Central Region has the highest prevalence of 2.7% among the five regions while Montserrado, Margibi, and Grand Bassa counties have the highest HIV prevalence among the 15 counties and together account for about 70% of the burden of disease in the country [6]. HIV predominantly affects vulnerable populations like commercial sex workers (CSW), men in same sex relationships, the military, young women and urban dwellers [5,6].

**Figure 1:**
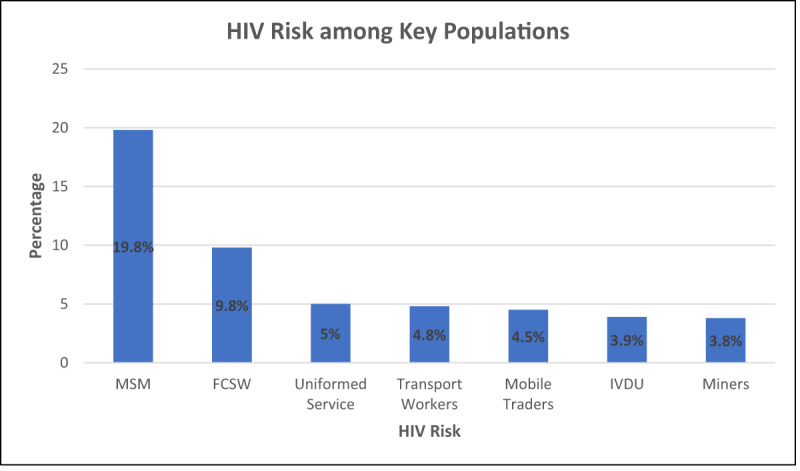
**Legend:** Prevalence of HIV among Key populations (Adapted with permission from the Republic of Liberia Country progress Report 2013, ref 7). **Abbreviations:** MSM – men who sleep with men, FCSW – female commercial sex workers, IVDU – Injection drug users.

In an Integrated Biological and Behavioral Surveillance Survey (IBBSS) from 2013, the most at-risk population was MSM (19.8%), followed by female CSW (9.8%), Uniform services personnel (5.0%), transport workers (4.8%) and mobile traders (4.5%), injection drug users (3.9%) and miners (3.8%) [6,7] (***Figure 1***).

## HIV in Liberia – national response

Since description of the first case of HIV in Liberia in 1986, the national response and control efforts have focused on scaling up HIV service delivery along the continuum of care – testing, enrollment and retention in care and achieving viral suppression [8]. However, protracted civil wars and the devastating 2014 to 2016 Ebola virus disease (EVD) outbreak posed serious challenges and slowed down the process of revamping the healthcare system [10,11] and created a shortage and maldistribution of the health workforce [12] with direct deleterious effects on HIV service delivery [13].

The National AIDS Commission (NAC) was the primary organization established in year 2000 to coordinate, monitor and mobilize resources for Liberia’s response to HIV and AIDS. Its mandates included promoting the active involvement of the private and public sector, in implementing HIV and AIDS services in Liberia. More recently, the Ministry of Health (MoH) through the National AIDS and Sexually Transmitted Infection (STI) Control Program (NACP) in collaboration with implementing partners (IPs) like the Resilient and Responsive Health System (RRHS) program partners and working with all County Health Teams (CHTs) are responsible for ensuring the provision of HIV services and adherence to National guideline for people living with HIV (PLWH) [8]. Based on the observation that the HIV program in Liberia struggles to deliver a high-quality client-centered health service that harmonizes responses for care, treatment and prevention needs, we analyze these challenges across the HIV continuum of care.

## Progress Towards UNAIDS 95-95-95 – Resources and Gaps

In 2014, the Joint United Nations Program on HIV/AIDS (UNAIDS) announced an ambitious FastTrack strategy to end AIDS as an epidemic by 2030. In the strategy, a 95-95-95 target was set, whereby, 95% of PLWH should know of their status, of these, 95% should be on antiretroviral therapy (ART), and 95% of people on treatment should have a fully suppressed viral load by 2030 [14]. Despite the adoption of this strategy since then [8], these targets have not been achieved in Liberia [8,15]. At the end of 2019, while the global HIV testing and care continuum stood at 81-67-59 [1], in the UNAIDS spectrum estimate for Liberia the first 95 was 57, the second 95 was 55 and for the third 95, there was no estimate [16].

Priorities for effective HIV service delivery have been grouped, according to the HIV care cascade, into: optimizing HIV testing, linkage from HIV testing to care; initiating antiretroviral therapy (ART); supporting adherence, retention, and reengagement in care as necessary; managing advanced HIV disease and its complications (AHD); access to viral load testing, comprehensive chronic disease care and service integration [17,18]. However, this cascade must be viewed through the overarching lens of fragile health systems within which the HIV program is situated including the interaction of the HIV pandemic with other existing chronic disease care models, the sustainable development goals [9,19] and more recently, the COVID-19 pandemic [1].

**Figure 2:**
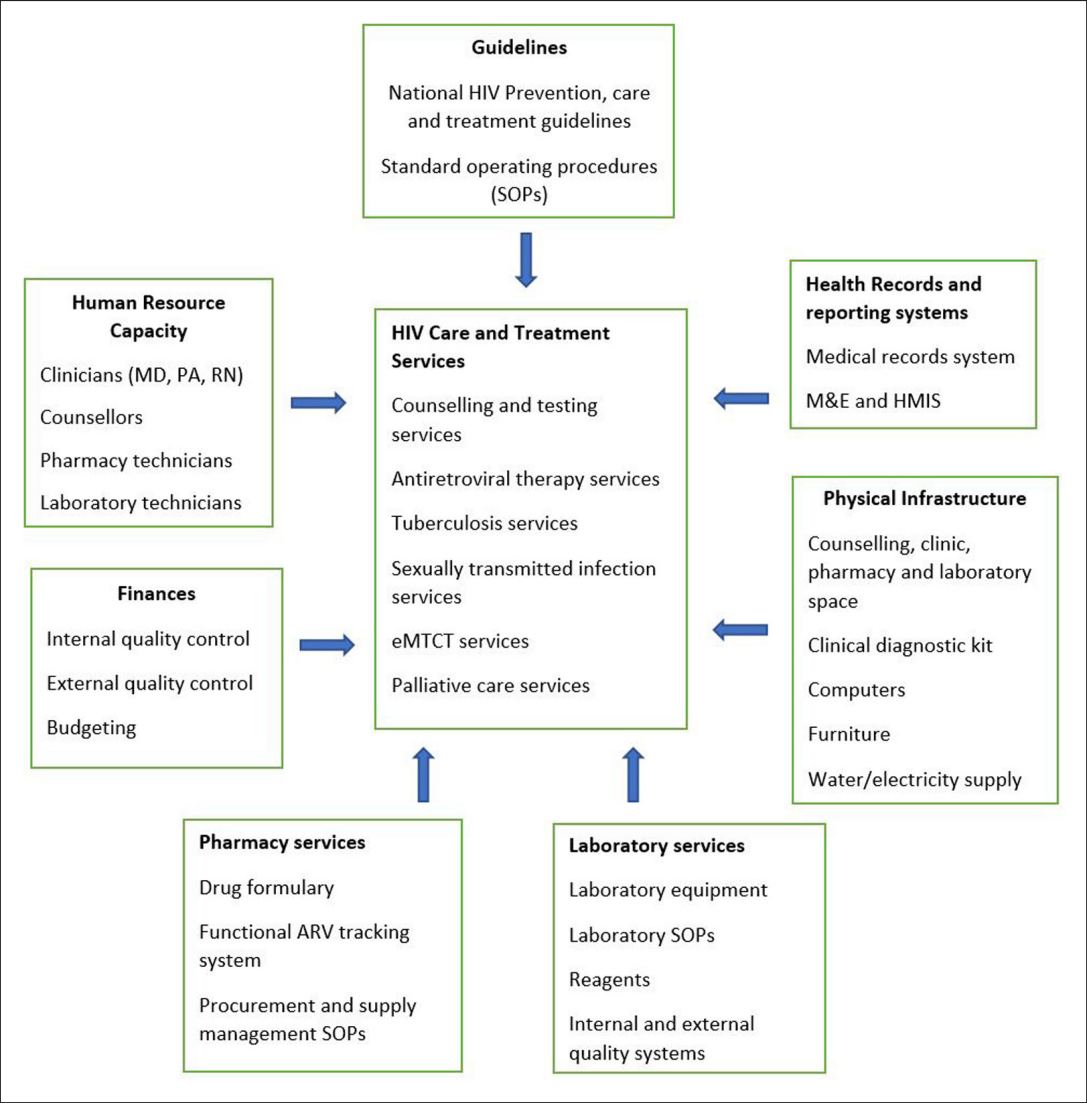
**Legend:** Organization of HIV/AIDS care service delivery within facilities in Liberia (Adapted with permission from the National guidelines for prevention, care and treatment of HIV and AIDS in Liberia 4th Edition). **Abbreviations:** MD – medical doctor, PA – physician assistant, RN – registered nurse, M & E – monitoring and evaluation, HIMS – health information management system, eMTCT – elimination of mother to child transmission.

In the face of the aforementioned challenges, to achieve resilience in HIV service delivery in Liberia, necessary components include: robust funding, guideline development in a national context for HIV testing services (HTS) and ART coverage, laboratory capacity and services, community engagement, supply chain management for drugs, testing supplies and commodities, addressing existing HIV inequities among subpopulations, eliminating stigma, data management systems and partners collaboration [17]. In addition, increasing programming needs for chronic care for comorbid diseases, bridging existing health disparities, and the engaging and growing of a skilled workforce to deliver high quality HIV care (***Figure 2***) are a sine qua non for overall health system strengthening [1,20,21].

In the last five years, overall domestic healthcare spending including for HIV services in Liberia has dropped [22]. Funding for HIV related activities is largely supported through external sources including the Global fund for AIDS, tuberculosis (TB) and malaria (GFATM), The US Presidents Emergency Fund for AIDS Relief (PEPFAR) and other international organizations through the MoH. In 2012, 99.2% of the total funds for HIV services were from external sources with domestic spending accounting for only 0.8% (with public funds accounted for 0.6% of the total funds while private funds accounted for 0.2%) [7]. Domestic funding for HIV services further dropped to 0.1% in 2015, a dismal amount compared to an 18% average for other low-income countries in the same bracket. This suggest that domestic spending on the HIV response has not been given a high priority [22] and this gap becomes glaring in the face of the magnitude of human resource and infrastructure needs of the HIV programs in Liberia which are not usually funded by donor organizations [23]. These have led to shortage of skilled medical providers leaving nurses and physician assistants to manage complex cases that would ordinarily require physician-level input, paucity of pediatric HIV providers and high staff attrition occurs due to poor remuneration and work conditions coupled with the significant loss of health personnel that occurred during the 2014 to 2016 EVD outbreak.

Despite resource and capacity challenges, certain programmatic successes and challenges are worth highlighting. HIV prevention, testing and treatment is based on WHO recommendations but adapted and contextualized to the Liberian setting [24,25] HIV testing services (HTS), prevention of mother to child transmission (PMTCT) and ART sites have been scaled up from 22, 3 and 3 sites respectively in 2006 to 585, 461 and 237 sites respectively in 2019, though the number of sites are still not adequate for effective coverage and adequate decentralization of care [8]. Supply-chain management systems have been instituted but intermittent stock-outs or expiration of testing and treatment supplies are not infrequent often related also to poor quantity forecasting and distribution [8].

Concerning community involvement and mobilization, task shifting of HIV-related services to community health assistants/workers (CHA/Ws) and PLWH network at the county level has been laudable but remain not fully integrated into the national testing and community ART distribution strategies [8] and disbursement of funds for the HIV response at the community level is still short of the recommended 30% [26]. Limited diagnostic capacity, lack of reagents and laboratory supplies, and lack of continuous electricity hamper routine monitoring of ART [8] while poor data quality and limited internet connectivity at county and district levels creates a bottleneck for reliable and timely use of health information for decision making [27,28]. These deficiencies will be reviewed further in the context of 95-95-95 targets.

### The First 95 (HIV Testing)

Timely and optimal HIV testing is the gateway to treatment with lifelong ART, preserving and prolonging life and decreasing community viral load (VL) to achieve epidemic control [28]. Knowledge of one’s HIV status helps HIV-negative individuals create opportunities to reduce their infection risk, and knowledge of HIV-positivity among HIV-positive individuals reduces HIV transmission risk. In the Liberia Demographic and Health Survey 2019 – 2020 (LDHS 2019 – 2020), regarding HIV testing services, among respondents aged 15–49 years in Liberia, only 72% of women and 65% of men knew where they could get an HIV test but more concerning was the finding that 66% of men and 45% of women had never been tested [29]. This falls far short of the projected first 95%.

Over the years, HIV testing sites have increased in Liberia but despite this improvement, challenges with delivering a high-quality HIV testing service remain. Uptake of HIV counselling and testing (HCT) is suboptimal and networks of PLWH are not actively engaged with testing outside of health facilities [8]. Within facilities, provider-initiated counseling and testing (PICT) and syndromic testing is the norm, universal testing is only applied to pregnant women. Rather than having a broad approach, community-based testing strategies have focused on key populations alone and rarely targeted the general population. Other barriers that have affected testing uptake include fear of a positive test result, low HIV risk perception, high level of stigma and discrimination, lack of confidentiality, and long waiting time [30,31].

Though community and facility based HTS is free in Liberia, the testing volume in 2018 was 269,426 with a positivity rate of 3.1% [26]. Of this number tested, 80% were women and 20% were men [3]. Women access HTS mostly during routine antenatal care for pregnancy which results in a higher testing volume compared to men where the low uptake of HTS services leads to a lower testing volume and a higher positivity rate and presentation with more advanced HIV disease at the time of diagnosis due to previously missed testing opportunities. In a Tanzanian study, reluctance to test for HIV among men was influenced by a masculinity ethos, which prevents them from expressing emotions in public and creates the notion that HIV testing is a woman’s domain as well as a heightened sense of risk related to extramarital relationships and resultant fear of receiving a positive diagnosis [31]. These factors may also be relevant issues in Liberia. Occasionally, stock-out of HIV testing kits due to supply chain issues including delays through port clearance and during last mile distribution to rural settings occur and this has been exacerbated during the COVID-19 pandemic [32,33]. The renewed emphasis on ensuring that HIV testing services are coupled to care, treatment and combination prevention services [17] are weak with delays in rapid ART initiation such that not all newly diagnosed PLWH get to receive lifesaving treatment on time.

### The Second 95 (ART Programs)

Effective combination antiretroviral therapy (cART) has resulted in significant reduction in HIV-related morbidity and mortality, near-normal life expectancy among PLWH, as well as preventing new HIV transmission [8]. The number of health facilities offering ART has increased substantially from three facilities in 2006 to 237 in 2019 resulting in a gradual increase ART coverage. By the end of December 2019, a total of 17,264 adults and children approximately 33% of PLWH in Liberia were on ART [16,34]. In 2013, a cohort study showed that 12–month retention among patients initiated on ART was 69.9% compared to 24.7% among patients not on ART. At 36 months, the retention rate fell further such that among ART patients, it was 48.7% compared to 9.4% among patients not on ART [7]. The common reasons for loss-to-follow up include transportation issues due to distance from facilities, self-transfer to other facilities, relocation, denial, resort to alternative medicine, fear of disclosure, conflict with work and drug side effects. it is expected that the shift to a test and treat strategy, adopted in 2016, will increase retention in care.

Generic fixed dose combination ARVs in Liberia are made available through HIV programs by The Global Fund to Fight AIDS TB and Malaria (GFATM) and PEPFAR after WHO prequalification [35,36]. Despite the financial investment in program processes and controls to scale up HIV activities by ensuring linkages to care and delivery of quality medicines and commodities to beneficiaries including key affected populations, the 2019 GFATM audit report for Liberia, rated these as partially effective [37]. The key issues identified were suboptimal financial management with gaps in recording, tracking and utilization of grant assets; poor supervision and oversight function and mentoring; as well as gaps in the national community health worker strategy leading to ineffective linkage in the HIV care cascade [7,8,38].

The National AIDS Control Program, in 2019, in line with WHO guidelines [24,25], transitioned from Efavirenz (EFV) to Dolutegravir (DTG)-based fixed dose regimen) along with 41 other low and middle-income countries (LMICs) to optimize ART [38] to achieve the UNAIDS treatment targets for HIV [1]. The DTG-based regimen – a WHO preferred first line regimen [38] achieves rapid virologic suppression, has a high resistance barrier, is well tolerated, and has a favorable drug-drug interaction profile [39].

While treatment availability and utilization have improved, there remains a significant challenge in the diagnosis and management of people presenting with advanced HIV disease (AHD), largely due to limited availability of diagnostics and treatment options for these preventable complications [40]. Examples include limited capacity to screen for, prevent or treat opportunistic infections such as tuberculosis and cryptococcal meningitis- both associated with high mortality rates when undiagnosed and untreated. Lack of CD4 testing makes suspicion of AHD challenging [40] and frequently results in late recognition of AIDS defining presentations.

Supply chain management, a critical piece of ART availability is being improved in Liberia. The USAID Global Health Supply Chain Program-Procurement and Supply Management (GHSC-PSM) project works with the Liberian National Drug Service (NDS) and Central Medical Store (CMS) to improve the availability of and access to life-saving HIV commodities [41]. Priority activities include efficient consolidation of warehousing at the central level, distribution, and supply chain technical assistance using data, and collaboration with key local stakeholders to strategically review supply chain operations and refine approaches for improved performance, increased efficiency, and country ownership [41]. In 2018, only 4.8% of ART sites had a stock-out of one or more required antiretroviral medicines during a defined period [26].

### Third 95 (Viral Suppression)

Achieving the individual and public health benefits of the UNAIDS 95-95-95 targets ultimately hinges on achieving the third 95 – community-wide viral load suppression [14]. The single most important method for reaching this target is expanding access to HIV ribonucleic acid (RNA) VL testing for monitoring response to ART and earlier detection of ART failure compared to reliance on clinical and immunological parameters [42,43]. Determination of “plasma” VL provides important information to providers and clients on the effectiveness of ART and provides information to countries regarding progress and gaps towards the target for epidemic control.

In 2013, the World Health Organization (WHO) recommended viral load testing six months after initiation of ART and every 12 months afterwards to detect treatment failure (defined as HIV RNA VL >1000 copies/mL on two consecutive measurements at least three months apart), and to guide switching to second line ART [44]. The guidelines stress that viral load testing should be performed when resources permit, and treatment should not be withheld if laboratory capabilities are not available. Liberia has struggled with capacity for VL testing [8], and currently, so few facilities offer VL that they are unable to cope with the testing needs and volume of the national program. In Liberia, like other resource limited settings, financial constraints; suboptimal installed capacity and frequent need to repair laboratory equipment; lack of reagents; insufficient and overburdened healthcare professionals, poor training of laboratory staff; weak transport and laboratory systems are all observed barriers to scaling up of VL testing in resource limited settings [45,46,47].

In 2020 following a needs assessment at one of the large volume ART site – John F Kennedy Medical Center (JFKMC) in Monrovia, a memorandum of understanding (MoU) was developed between the NACP, Partnership for Research on Vaccines and Infectious Diseases in Liberia (PREVAIL) and JFKMC to create access to GeneXpert platforms and other research infrastructure [15]. This system has added additional capacity for 120 VL tests per week significantly ramping up existing VL testing capacity.

Recently, education of PLWH has increased the awareness and created a demand for VL testing in the Liberian HIV program. However, with the absence of electronic health records (EHR), medical records and data management within the HIV program are still paper based, requiring cumbersome clinical processes to trigger or predict due dates for VL testing. Timely availability, reliability and completeness of data has created challenges with improving the VL testing processes and continuous quality improvement (CQI). Observational evidence across HIV programs in sub-Saharan African suggests that VL testing rate is better among countries with EHR systems. On-site phlebotomy, absent in Liberia HIV programs, may also improve testing rates [45]. There are currently no facilities for routine ARV drug resistance testing in Liberia to inform second and third line ART selection.

## Opportunities and Recommendations for Optimizing HIV-Related Services in Liberia

Falling short of the UNAIDS targets for 2020 [1], all stakeholders must come together to plan and align activities to address the many gaps in HIV service delivery across the continuum of care in Liberia taking advantage of the renewed interest in achieving the 95-95-95 targets for 2030 by working towards the UNAIDS interim targets for 2025 [48]. The NAC and NACP must harness current evidence-based recommendations from WHO and streamline it with the Liberia National strategic plan for HIV 2015 to 2020 [49]. It should be recognized that the HIV response is coupled with the wider effort to end poverty, fulfill the right to health and other human rights and goals within the agenda for Sustainable Development [19,48]. Lessons and experiences garnered from programmatic activities in high volume facilities should be harnessed and cascaded down to peripheral facilities putting PLWH and communities at risk at the center of the agenda.

### Combination Prevention Services

Under the leadership of the MoH and the NAC, a Fast-Track plan for 2019–2020 was developed that identifies high-impact programs, and barriers that must be removed to ensure better service delivery. It also recognizes that prevention measures must be escalated and that stigma and discrimination must be reduced [3].

Innovative combination approach to HIV prevention that includes behavioural, biomedical and structural approaches and tailored to those in greatest need are needed to reach people with undiagnosed HIV earlier and improve the first 95. Budgets for condom and social marketing programs should be reinstated targeting a new generation of sexually active young people that have not been exposed to the intense condom promotion that was in place a decade ago in view of the prevailing poor knowledge of combination prevention [29] and the need for youth-focused HIV efforts in Liberia to address risk behavior such as transactional sex, multiple and concurrent sexual partnerships and how to negotiate safe sex [50,51]. The introduction of pre-exposure prophylaxis (PrEP) will contribute to steeper reductions in HIV infections among high risk groups.

### Linkage from HIV Testing to Care and Rapid ART Initiation

The Liberia catch-up plan seeks to triple the country’s test and treat figures and link people with positive tests to prompt treatment. Traditional testing strategies should be combined with newer testing strategies like the HIV self-testing and universal testing. The catch-up plan will be guided by a location–population approach, with a service delivery bias towards the three counties of Montserrado, Margibi, and Grand Bassa with the highest unmet need for HIV testing, treatment and care services, urban areas and some other locations with a focus on adults aged 15–49 years, pregnant women, MSM, commercial sex workers, people who inject drugs, prisoners, mine workers and other men – declared by UNAIDS as a “HIV blind spot” who traditionally have poor uptake of testing and other HIV services [3].

Rapid ART initiation with newer and potent medications like DTG within seven days of HIV diagnosis including same day ART initiation will lead to improved retention in care and increased viral suppression [19,52]. Concerns regarding the possible risk of immune reconstitution inflammatory syndrome (IRIS) among people with advanced immunosuppression starting ART may lead to a delay. To avoid this risk, baseline screening services for AHD need to be expanded. These should include reintroduction of routine CD4 count testing of ≤200 lells/μL to diagnose AHD and other tests in the package of care to rapidly identify and treat, or exclude, active TB or cryptococcal disease without delaying ART initiation for most patients [41].

### Training, Task Sharing and Decentralization

There is need to strengthen the frequency of training and continuing professional development (CPD) for health workers in the counties on advances in HIV care. Content should be tailored to target priority areas like task sharing protocols with clear roles and responsibilities delineated to guide clinical care and create clear pathways for physician oversight or referral especially with pediatric ART, elderly patients, newly diagnosed cases, adverse drug reactions, switching to second or third line ART, managing NCDs, inpatient care and AHD care [17]. Other priority areas include training of laboratory staff who are usually missing from trainings. North south collaborations with partner organizations like PEPFAR-funded Resilient and Responsive Health System (RRHS) partners, who are supporting HIV care and, health workforce training using innovative approaches and overall health system improvement in Liberia should be strengthened. These training and task sharing models can also be replicated in the counties as part of a decentralized HIV service delivery to bring care to where people live.

### Sustaining ART, Retention in Care and Viral Suppression

There is emerging evidence that ART can be successfully and safely started outside of health facilities following community-based testing [53]. This approach improves access to treatment for populations that may have difficulty accessing traditional health services and is associated with high acceptance and readiness for same day ART and high VL suppression [52]. This out-of-facility ART initiation may be another promising intervention to optimize the impact of test and treat programs [19].

In 2016, WHO recommended providing three to six months multi-month dosing (MMD) for stable clients to reduce the frequency of clinic visits and ART refills [25]. This approach to differentiated service delivery (DSD) was widely adopted across facilities during the 2020 COVID-19 lockdown [33]. It is supported by major funding agencies including PEPFAR, International AIDS Society (IAS) and the GFATM and has been incorporated into the national guidelines [33]. Pharmacy delivery [55] needs to be scaled up to expand access in a client centered approach that simplifies and adapts HIV services across the cascade in ways that both serve the needs of subpopulations of PLWH better and reduce unnecessary burdens on the health system [54].

There are multiple reasons for suboptimal adherence, disengagement from care and loss to follow up. A combination of supportive interventions including use of peer or lay counselors, participation in adherence clubs, use of mobile text messaging and reminders are useful to support adherence and retention in care [1,3,25]. After initiation of ART, follow up calls within the first two weeks can be helpful to enhance return. These can be coupled with interventions that reduce stigma and discrimination. Pharmacy refill records are a feasible and reliable approach to assess adherence and should be optimized and utilized to improve adherence, retention and VL suppression [17]. VL testing and suppression can be improved by the use of EHR in the HIV clinics to enhance identification of clients and due date for VL testing, integrating phlebotomy services in HIV clinics and reducing turnaround time for VL results including prompt communication of high VL results to clients using text messaging or phone calls which will enhance prompt switching to more potent second or third line ARVs.

### Managing Advanced HIV Disease and Integrated Services

Because of health workforce shortages, the Liberian HIV program is built on non-physician health care providers. There are existing gaps in delivering quality management of AHD and NCDs and this must be prioritized. Task sharing protocols must be enhanced to create clear and timely referral pathways that FastTrack and connect PLWH with AHD to physicians for inpatient care especially within the high volume and tertiary facilities like JFKMC and RDH where specialist physician providers are available. Funding should be provided to procure CD4 tests, GeneXpert platforms for HIV VL, urinary LAM and Serum CrAg to deliver the WHO minimum package of care for AHD [41]. Screening for diabetes and hypertension (common NCDs) should become routine and care for PLWH with these conditions should be integrated into the HIV program as part of a comprehensive package of care [55].

### Research

Beyond routine reporting of HIV data to the health information management systems, effort should be made to enhance and capture good quality data from routine programmatic activities for quality improvement and research [15]. A good example is the leadership and management structure and quality management committee set up at the JFKMC by the RRHS programme faculty. These systems should be strengthened to collect timely, reliable and complete data that can be utilized for quick decision making and enhancing quality of care. This should be in addition to conducting Liberia-context specific operational research on current HIV program challenges including: the best methods for ART initiation and retention in care, impact of transition to DTG on VL suppression, drug resistance patterns, burden of NCDs among PLWH, DSD models and PrEP, to inform policy and guidelines as well as clinical practice ultimately towards addressing and improving the overall quality of the health system and HIV service delivery in Liberia.

## Conclusion

Despite the existing gaps and challenges with the health system and HIV service delivery in Liberia, opportunities abound to improve quality of care towards achieving the 95-95-95 targets by 2030. This would require coordination, commitment and resolve of all stakeholders–health care providers, program managers, international partners/donors and government–to provide resources, implement programs and adopt innovative solutions to address the many opportunities highlighted. Once accomplished, PLWH in Liberia will achieve the so called fourth “90”–to live a long and healthy life and collectively achieve epidemic control.
